# Navigating the Professional Journey for Adults with Attention Deficit/Hyperactivity Disorder: Challenges and Strategies

**DOI:** 10.1192/j.eurpsy.2024.478

**Published:** 2024-08-27

**Authors:** S. A. Pinho, F. Coutinho

**Affiliations:** Hospital de Magalhães Lemos, Centro Hospitalar Universitário de Santo António, Porto, Portugal

## Abstract

**Introduction:**

Attention deficit/hyperactivity disorder (ADHD) is a neurodevelopmental disorder that affects approximately 5% of adults. Individuals with ADHD often display symptoms of inattention, including poor time management and difficulty concentrating and completing tasks. Hyperactivity frequently attenuates over time and transforms into inner restlessness, leading to workaholic behaviors. Impulsive symptoms, on the other hand, may manifest as irritability and low frustration tolerance.

**Objectives:**

To describe the workplace challenges that adults with ADHD face and to explore strategies to improve their occupational outcomes.

**Methods:**

A non-systematic review of the clinical literature available in PubMed was conducted using the keywords: “employment” and “attention deficit hyperactivity disorder”.

**Results:**

Individuals diagnosed with ADHD, in contrast to those without the condition, statistically exhibit poorer job performance and increased lateness, job instability, workplace injuries, particularly traffic accidents, comorbid diseases, and financial problems. Therefore, they often work harder to compensate for their limitations however the findings regarding the health impact of such high job demands are inconsistent. Stimulant therapy during childhood is the main predictor of successful adult employment. Contrarily, risk factors for workplace impairment in ADHD include female gender, executive deficits, lower IQ, less education, combined/inattentive subtype, and history of substance abuse, depression, or anxiety. It was also demonstrated that ADHD individuals may thrive in manual and creative roles and hyperactivity can benefit self-employment. Psychiatrists should offer psychoeducation, along with psychostimulants if necessary, as it is the first-line treatment. Nonetheless, the long-term impact of pharmacological treatment on professional outcomes remains unclear. Although most employers lack ADHD knowledge, workplace strategies including well-defined duties, feedback, job control, and flexibility have been shown to effectively mitigate ADHD symptoms.

**Conclusions:**

Evidence suggests that a significant amount of employees with ADHD face challenges in finding and keeping a job. Thus, identifying and treating ADHD in adulthood is imperative to help them selecting careers that align with their strengths and weaknesses, which are partially influenced by ADHD, and to promote optimal occupational health. This effort requires collaboration between psychiatry and occupational health professionals. Additionally, it is necessary to start implementing educational campaigns among workforce teams to effectively accommodate workers with ADHD. Further studies are needed to develop occupational programs and rehabilitating interventions tailored to this population.

**Disclosure of Interest:**

None Declared

